# Compensatory Evolution of Intrinsic Transcription Terminators in *Bacillus Cereus*

**DOI:** 10.1093/gbe/evw295

**Published:** 2017-02-14

**Authors:** Ksenia R. Safina, Andrey A. Mironov, Georgii A. Bazykin

**Affiliations:** 1 Sector for Molecular Evolution, Institute of Information Transmission Problems (Kharkevich Institute) of the Russian Academy of Sciences, Moscow, Russia; 2 Department of Bioengineering and Bioinformatics, Lomonosov Moscow State University, Moscow, Russia; 3 Center for Data-Intensive Biomedicine and Biotechnology, Skolkovo Institute of Science and Technology, Skolkovo, Russia

**Keywords:** intrinsic transcription terminators, GU wobble pair, compensatory evolution

## Abstract

Many RNA molecules possess complicated secondary structure critical to their function. Mutations in double-helical regions of RNA may disrupt Watson–Crick (WC) interactions causing structure destabilization or even complete loss of function. Such disruption can be compensated by another mutation restoring base pairing, as has been shown for mRNA, rRNA and tRNA. Here, we investigate the evolution of intrinsic transcription terminators between closely related strains of *Bacillus cereus*. While the terminator structure is maintained by strong natural selection, as evidenced by the low frequency of disrupting mutations, we observe multiple instances of pairs of disrupting-compensating mutations in RNA structure stems. Such two-step switches between different WC pairs occur very fast, consistent with the low fitness conferred by the intermediate non-WC variant. Still, they are not instantaneous, and probably involve transient fixation of the intermediate variant. The GU wobble pair is the most frequent intermediate, and remains fixed longer than other intermediates, consistent with its less disruptive effect on the RNA structure. Double switches involving non-GU intermediates are more frequent at the ends of RNA stems, probably because they are associated with smaller fitness loss. Together, these results show that the fitness landscape of bacterial transcription terminators is rather rugged, but that the fitness valleys associated with unpaired stem nucleotides are rather shallow, facilitating evolution.

## Introduction

Because evolution mainly proceeds through small mutational steps, it requires the existence of contiguous pathways in the genotypic space traversing sufficiently fit genotypes. Adaptive landscapes have a huge dimensionality, and their shape is generally unknown. Still, their local properties can be studied, for example, by analyzing macroevolutionary patterns (De Visser and Krug 2014; Bazykin 2015). Of particular interest is the frequency and the mechanism of crossing of fitness valleys—evolutionary events involving multiple mutations some of which are deleterious. Such events may or may not involve transient fixation of deleterious single mutants; in either case, the subsequent fixation of the double mutant typically occurs fast (Kimura 1985; Stephan 1996; Bazykin et al. 2004).

The fitness landscape of proteins is determined by a network of structural and functional interactions between positions, and is generally complex (Podgornaia and Laub 2015). The fitness landscape of functional RNA is simpler. Unlike DNA, RNA is a single-stranded molecule. Yet, it can be folded into a wide variety of three-dimensional structures containing double-stranded regions, determining the stability of the molecule and defining its function. In different RNA types, mutations can disrupt Watson–Crick (WC) interactions causing structure destabilization and/or change or disruption of function (Elson et al. 2009; López de Quinto and Martínez-Salas 2000). However, RNA structure and function can be restored by another mutation, which compensates the disrupted interaction. For example, in mammalian mitochondrial tRNAs, compensation for pathogenic mutations may proceed through different mechanisms, including restoration of the broken WC pair, formation of a new pair or strengthening of an existing pair (Kern and Kondrashov 2004). In particular, two-step AU ⇊ GC switches proceed through strongly deleterious variants and thus involve crossing deep fitness valleys (Meer et al. 2010). In a range of systems, including tRNA (Meer et al. 2010), rRNA (Rousset et al. 1991), and viral RNA (Assis 2014), such double substitutions in helical regions preferentially occur via the GU base pair.

Here, we study compensatory evolution of bacterial intrinsic transcription terminators. Bacteria utilize two different mechanisms for transcription termination. Rho-dependent termination involves Rho-factor, a helicase that unwinds the RNA–DNA hybrid duplex releasing the mRNA (Ciampi 2006). In rho-independent (intrinsic) termination, the newly synthesized transcript forms a hairpin followed by an oligouridine tract. The hairpin disrupts the transcriptional bubble and the hybrid duplex and blocks elongation, while the oligouridine tract destabilizes the RNA–DNA duplex and lowers elongation efficiency. Acting together, these elements cause RNA polymerase arrest and mRNA release (Datta and von Hippel 2008). Hence, the RNA hairpin is critical for transcription termination and is expected to be conserved. Conservation of the secondary structure can be attainable even as the nucleotide sequence changes if base pairing is preserved; any single-nucleotide change in the hairpin by itself may be expected to be more or less disruptive, but compensable by additional changes. Such compensating substitutions may be expected to occur fast, so that base pair complementarity is preserved most of the time. To study this process in detail, we analyzed the compensatory substitutions in rho-independent transcription terminators of *Bacillus* *cereus.*

## Material and Methods

### Phylogenetic Tree Reconstruction

We downloaded from GenBank the 25 complete annotated Refseq genomes of *B. cereus* available to date (accession codes: NC_012472, NC_014335, NC_018491, NC_003909, NC_011658, NC_011773, NC_011772, NZ_CP009941, NC_011725, NC_004722, NZ_CP009628, NZ_CP009318, NC_016779, NC_016771, NC_006274, NC_011969, NZ_CP009968, NZ_CP012483, NZ_CP009686, NZ_CP009596, NZ_CP009605, NZ_CP009641, NZ_CP009369, NZ_CP009590, and NZ_CP009300) and *B. cytotoxicus NVH 391-98* (accession code NC_009674) as the outgroup species.

We obtained groups of orthologs for these 26 genomes using OrthoMCL (Li 2003), for a total of 2,473 groups containing exactly one ortholog per genome. We then aligned these orthologs with Muscle (Edgar 2004) and concatenated alignments for which sum-of-pairs scores normalized by the alignment length were greater than 45 into one superalignment with a total sequence length of 1.95 Mb. This alignment was filtered by Gblocks (Talavera and Castresana 2007) with ‘With half’ gap treating option, leaving 1.94 Mb.

On the basis of this alignment, we reconstructed the phylogenetic tree under the GTR+Γ model using RaxML (Stamatakis 2006), rooted with *B. cytotoxicus NVH 391-98* (supplementary fig. S1, Supplementary Material online). Bootstrap iterations (10,000) were performed to estimate branch support.

### Terminator Sequences Retrieval

Independently, we used OrthoMCL to obtain groups of orthologs for the 25 genomes of *B. cereus*, and aligned them with muscle. We discarded those genes in which the alignment started with a gap of length 200 or above, or ended with a gap of 50 or above, in one or more of the strains, because in such genes, the alignment of the predicted orthologous terminator tended to be poor. This left 3,093 genes. For each of these genes, we extracted the regions between position 30 upstream and position 180 downstream of the stop codon (“downstream regions”).

We then focused on the 1,687 of these genes for which the positions of terminator sequences have been predicted in the *B.* *cereus ATCC 14579* genome (AC NC_004722; hereafter, reference genome) (De Hoon et al. 2005); http://bonsai.hgc.jp/∼mdehoon/terminators/NC_004722.trms; last accessed January 19, 2017) and fell into our analyzed downstream region. Using these predicted positions of terminator stems, we then resolved stem structures in this genome using RNAfold tool from the Vienna package (options -noLP, -C; Lorenz et al. 2011). We employed this two-step procedure because existing prediction tools either do not output the exact stem structure (De Hoon et al. 2005; Kingsford et al. 2007) or utilize simplistic algorithms; for example, WebGester (Mitra et al. 2011) does not consider poly-U tract quality during the prediction process, while the TransTermHP (Kingsford et al. 2007) estimates free energy of stems using Nussinov algorithm and ignores stacking energies. Finally, we excluded terminators with stems shorter than 5 or longer than 19 base pairs, leaving a total of 1,605 predicted terminator sequences in the reference genome.

### Multiple Alignment of Terminator Sequences

To create a structure-aware alignment for the orthologous terminator sequences of *Bacillus cereus* genomes, we used the following procedure. First, we used Clustalw (Thompson et al. 1994) to align each reference terminator sequence inferred at the previous step to the orthologous downstream region in each non-reference genome. The resulting sets of best-hit regions together with 10 flanking nucleotides from each side were used for RNA multiple structural alignment with MAFFT X-INS-I (Katoh and Toh 2008). We transferred the RNA structure obtained with RNAfold for the terminator from the reference genome to the other sequences and defined a pair of interacting alignment columns as a pair of columns that correspond to the interacting positions in the reference genome. We then retained only those alignments in which each of the aligned sequences met all of the following conditions: (i) possessed RNA stem energy of at least −5 kcal/mol, (ii) contained at least five complementary (WC or wobble GU) nucleotide pairs in interacting columns, (iii) contained complementary pairs in at least 70% of pairs of interacting columns, and (iv) contained no more than 30% of unpaired nucleotides both in the left and the right parts of the stem, leaving 1,116 alignments. Finally, we calculated pairwise nucleotide distances between the reference sequence and each other sequence and excluded the 189 additional genes in which any of the 24 sequence identity values was below 70%, leaving 927 alignments.

In these alignments, we discarded those few pairs of interacting columns in which the most common pair was not WC or GU. Finally, we manually curated the alignments containing pairs of polymorphic interacting columns, since such alignments are the most dubious. This left 286 column pairs in 202 alignments, out of the 302 considered column pairs in 208 alignments.

### Prediction of Bidirectional Terminators

Although bidirectional terminators can be predicted most reliably only experimentally (De Hoon et al. 2005), they can also be predicted computationally. We classified pairs of adjacent genes in the reference genome as cooriented (end-to-head) or convergent (end-to-end). For convergent pairs, we checked whether each of them had a predicted terminator (De Hoon et al. 2005). If terminators were predicted for both genes, we checked if they coincided (forming the same stem structure on the opposite strands). If a terminator was predicted for only one of the two genes, we checked if its reverse complement could potentially form a terminator structure and satisfied our conditions for it. Several such pairs of genes were excluded because the terminator for the second gene could not be predicted reliably, as the reverse complement of the predicted terminator did not form a potential terminator structure (2 genes), was located too far apart from the second gene (26 cases), or was located inside the second gene (3 cases). For the coinciding terminators, each pair of interacting alignment columns was counted independently for each gene (and therefore, for each strand). Using two-tailed Fisher’s exact test, we tested whether the fraction of positions with substitutions was different for convergent genes, compared with cooriented genes.

### Ancestral States Reconstruction and Analysis

For each alignment column in each of the 927 alignments (202 of which contain pairs of polymorphic columns), we used PAML (Yang 1997); baseml, F81 model, assuming global molecular clock) to reconstruct the ancestral states at all internal nodes of the phylogenetic tree, assuming the tree topology reconstructed previously; results were similar when two other substitution models, JC (Jukes and Cantor 1969) and REV (Tavaré 1986) were used. Nodes with the PAML-reconstructed posterior probability of the ancestral state below 0.8 were considered irresolvable.

The expected fractions of cases in which both nucleotides in a WC pair were substituted were calculated by assuming that the two substitutions are independent. Specifically, for a given pair of nucleotides *A* and *B*, the probability of *A* to change was calculated as the number of cases with the substituted *A*, divided by the total number of ancestral *AB* pairs. The expectations for *B* were calculated similarly, and the expected fraction of two substitution cases equaled the product of these two values.

**Fig. 1.—:**
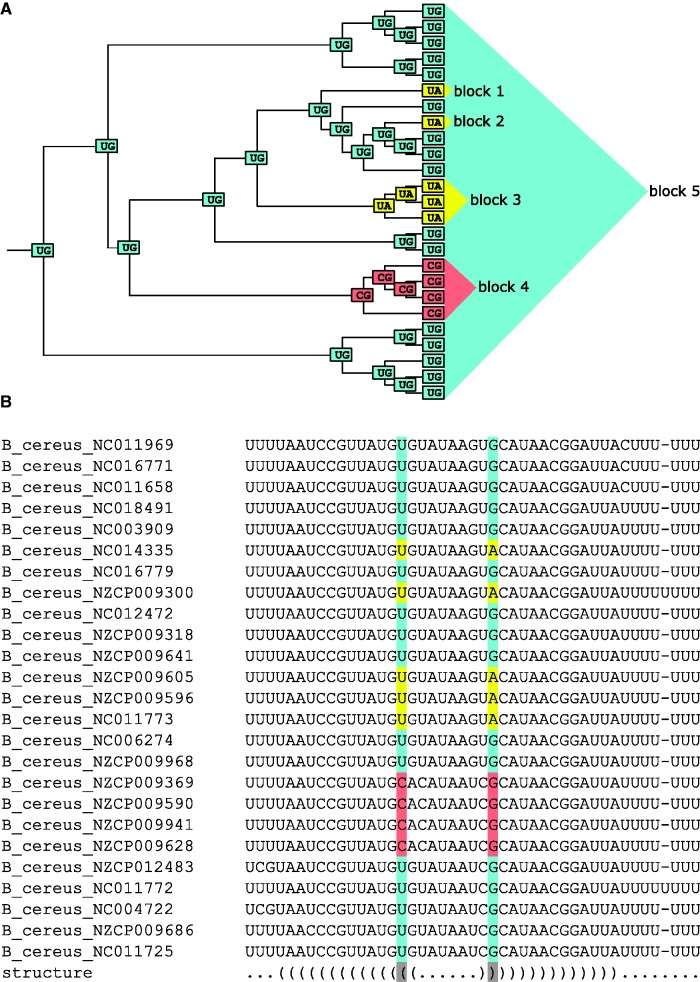
Identification of phylogenetic blocks in the transcription terminator of locus BC0230 (*LacI* transcription factor). *A*, A schematic representation of the phylogenetic tree of BC0230, with the tags in nodes corresponding to the nucleotides at two interacting positions of the hairpin. Different blocks on the tree are colored differently and denoted as triangles. Block pairs 1–4, 2–4, and 3–4 represent base pair switches. *B*, The alignment of the BC0230 terminator, with the strains in the same order as in *A*. The last line represents the structure in the bracket notation. For the reference strain, sequence presented here corresponds to the (205,668–205,712) region of its genome.

Using the reconstructed ancestral states, for each nucleotide change, we tried to infer the branch of the phylogenetic tree where this change had occurred. For WC pairs of nucleotides involved in pairwise interactions, we then inferred all base pair switches. To do this, we employed the following algorithm. For each pair of interacting alignment columns, we defined “blocks.” A block is a contiguous group of nodes on the tree carrying the same state in both columns that are separated from the rest of the tree with a substitution in at least one of the columns (fig. 1). By iterating through all pairs of blocks carrying WC states (AU or GC), we then selected pairs separated by exactly one substitution at each of the two positions. This produced two lists of extant genomes separated from each other by exactly two substitutions, that is, that had experienced a base pair switch.

**Fig. 2.—:**
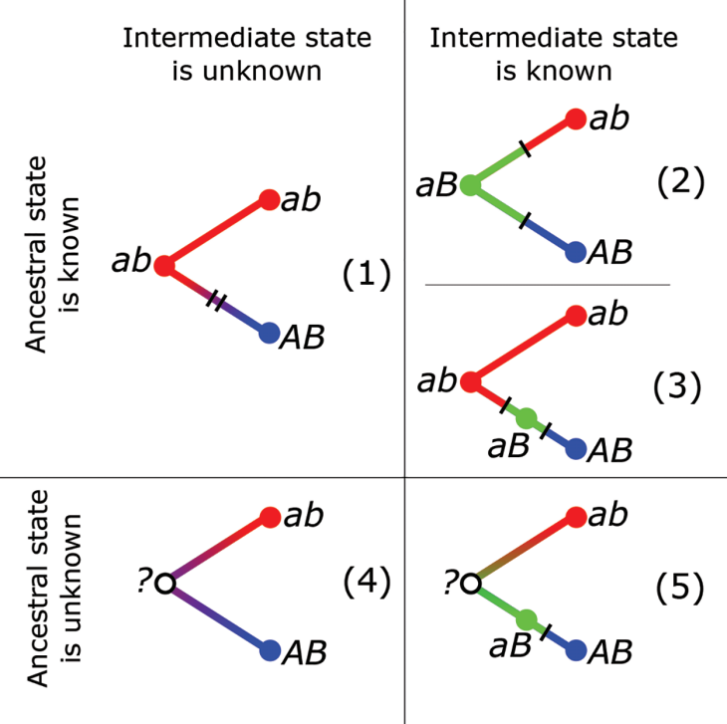
Classification of base pair switches by type (see text). Differing WC pairs are colored in red and blue, and the intermediate state, if known, in green. Unknown states are denoted with white circles. *a* and *b* correspond to the two nucleotides in an interacting pair, with the two states denoted by small and capital letters.

**Fig. 3.—:**
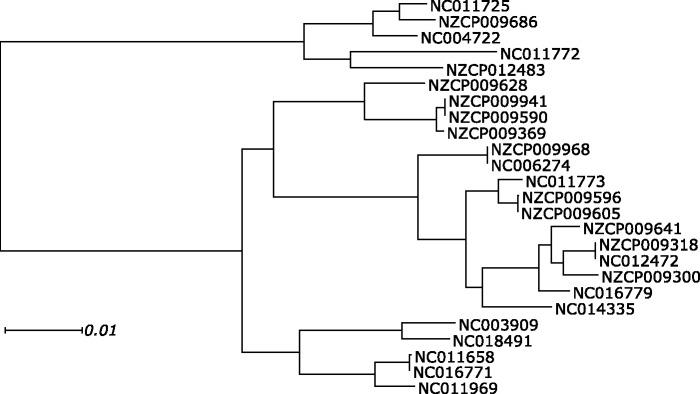
—Rooted phylogenetic tree reconstructed for the 25 *Bacillus cereus* genomes. All bootstrap values are 100%.

**Fig. 4.—:**
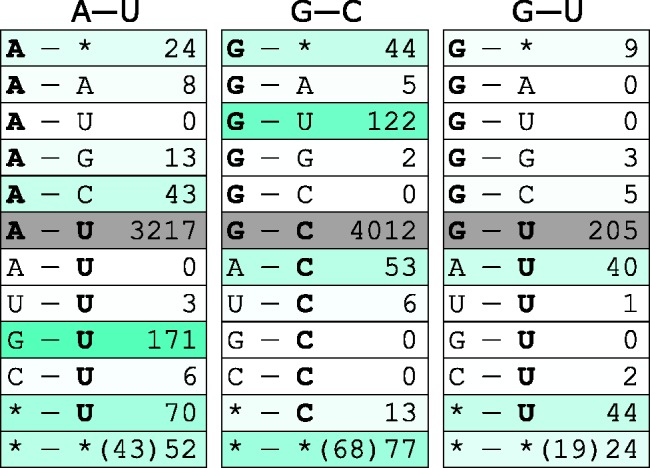
Nucleotide changes at interacting pairs of columns. For ancestral states A-U, G-C, or G-U, the values in rectangles show the number of pairs where each nucleotide was invariant in all compared strains (gray), or one or both nucleotides where variable (cyan colorscale, with darker colors corresponding to more frequent events; the invariant nucleotide is in bold). Asterisk in one of the columns corresponds to multiple substitutions in the same position. Asterisks in both columns correspond to substitutions (either single or multiple) in both positions, with the value in brackets showing the number of those pairs where a WC switch was observed.

**Fig. 5.—:**
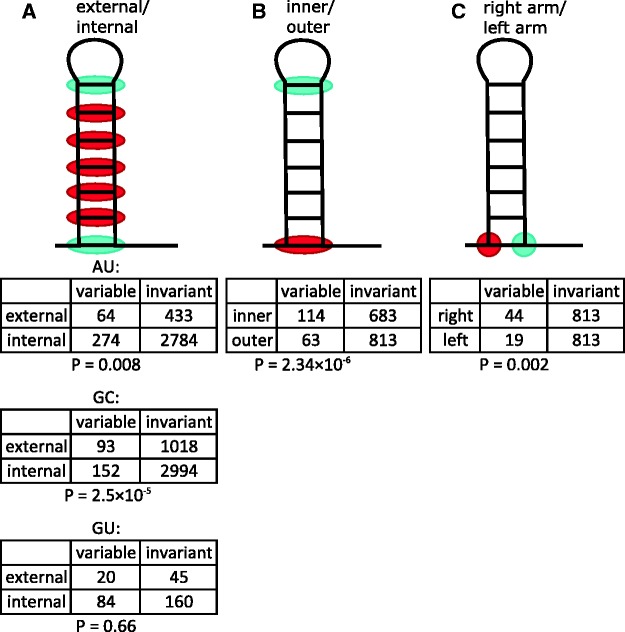
Frequencies of variable and invariant sites at different positions of the terminator stems. *A*, external vs. internal positions of the stem; *B*, inner vs. outer position of the stem; *C*, right vs. left arm of the outer position of the stem. Cyan, the category with an excess of substitutions; red, the complementary category. In *B* and *C*, AU, GC, and GU ancestral base pairs are pooled together. Tables contain the number of interacting columns where both nucleotides were invariant (“invariant”), or one of the nucleotides was variable (“variable”), in each of the two compared categories, with two-tailed Fisher's test *P*-value corresponding to each table.

We then classified each switch as belonging to one of the five switch types, depending on whether the ancestral and the intermediate state could be resolved (fig. 2). If the ancestral state was resolved, there were three scenarios for a switch between two WC pairs *ab* and *AB:* the last common ancestor could carry either of the two terminal WC states (*ab* or *AB*), so that both substitutions happened along the same lineage, and no nodes carried the intermediate state (fig. 2, 1); the last common ancestor carried the non-WC state *aB* or *Ab*, with one substitution occurring in each of the descendant branches (fig. 2, 2); or the last common ancestor carried the WC state, but a non-WC intermediate state, *aB* or *Ab*, was reconstructed at one of the intermediate nodes (fig. 2, 3). When the ancestral state was irresolvable, the intermediate state could either be never observed (fig. 2, 4) or reconstructed at one of the internal nodes (fig. 2, 5).

### Permutations

The phylogenetic distribution of events depends on the tree shape. To control for this, we generated 100,000 artificial column pairs by picking two columns randomly from the 572 polymorphic columns with reconstructed ancestral states and analyzed similarly to the pairs of interacting columns. These permutations were used to obtain the null distribution for the statistics used to analyze the phylogenetic positioning of substitutions; a statistic was considered significant if it fell beyond the 2.5% (or 97.5%) percentile of the expected distribution. All details of statistical analyses are provided in supplementary material S2, Supplementary Material online; supplementary materials S3 and S4, Supplementary Material online provide data on the filtered subset of 927 genes and the R-script performing all the analyses, respectively.

## Results

### Selection on Terminators Structure

Filtering of the initial set of 3,138 terminators predicted in the reference *B. cereus* genome resulted in 927 terminator sequences that were analyzed further. The length distributions of the predicted hairpins, loops and U-tracts (supplementary fig. S2, Supplementary Material online) were similar to those obtained experimentally (De Hoon et al. 2005).

We studied selection on RNA structure by analyzing conservation of the nucleotide sequence between closely related *B. cereus* genomes. Overall, the conservation of terminator stems is high: 90.72% of the alignment columns corresponding to hairpin stems are invariant, which is more than in other regions within the terminators (62.37% of the columns corresponding to loops, and 78.61% of the columns corresponding to poly-U regions) and in fourfold degenerate sites of protein-coding regions (63.46%), indicating strong selection acting on terminator structure. Moreover, when a column was not conserved, it was more frequently (96.37%) due to mismatch(es), and less frequently, to gap(s), compared with loops (82.86%) and poly-U regions (70.90%).

The pairs of stem columns annotated as interacting could carry one of the three pairs of nucleotides in the reference genome: AU (43.47%), GC (51.55%), or GU (4.98%). The majority of these pairs were invariable, including the GU pair that comprises 2.76% of all invariable column pairs.

Still, 8.18% of the columns involved in interactions are variable. Using the reconstructed phylogeny (fig. 3), we inferred the ancestral states for the terminator nucleotides, and used these data to understand how the stem regions evolved between the *B. cereus* strains (fig. 4). In 96.83% of all pairs of stem nucleotides where the state in the last common ancestor (LCA) of all *B. cereus* strains could be inferred, this state was a WC pair: AU (43.58%) or GC (52.37%); additionally, 4.02% of them were wobble GU pairs, with the remaining 0.02% being other non-WC pairs. Ancestral GU pairs were substantially more likely to experience a substitution in one of the nucleotides (31.23% of all GU pairs) than AU (9.37%) or GC (5.65%) pairs. Conversely, most of the substitutions in AU and GC pairs gave rise to the wobble pair GU, indicating that this substitution is associated with less, if any, fitness loss. AU → GU substitutions were slightly more frequent than GC → GU substitutions; reciprocally, most of the substitutions in the GU pair gave rise to AU. Both nucleotides in a pair were substituted in only 1.44% of AU, 1.78% of GC and 7.21% of GU pairs; still, this is much more than expected (0.32%, *P* = 4.0 × 10^−^^18^; 0.20%, *P* = 7.2 × 10^−^^46^; and 4.10%, *P* = 0.008, respectively; two-tailed binomial test) if the two substitutions had occurred independently (fig. 4).

In the majority of the cases where stem nucleotides have changed, only one of the two nucleotides in a WC pair has been substituted (86.67% of cases for AU, 76.09% of cases for GC, and 81.25% of cases for GU; fig. 4). Focusing on these cases, we asked where these variable nucleotides were located in the stem structure. Nucleotide belonging to AU and GC ancestral pairs, although not to GU pairs, were more likely to be variable when they were at external positions of the stem (i.e., the first and the last nucleotides of the stem), compared with when they were at internal positions of the stem (i.e., all other nucleotides; AU: 12.88% of external vs. 8.96% of internal, *P* = 0.008; GC: 8.37% of external vs. 4.83% of internal, *P* = 2.5 × 10 ^−^ ^5^; GU: 30.77% of external vs. 34.43% of internal, *P* = 0.66, Fisher's exact test), indicating that mutations disrupting base pairing at external positions are under weaker selection (fig. 5). Furthermore, among external positions, substitutions were more frequent at the inner positions (*P* = 2.34 × 10 ^−^ ^6^) than at the outer positions of the hairpin. Unexpectedly, among the outer positions, substitutions were slightly more frequent at the right arm, i.e., closer to the poly-U tract (*P* = 0.002), compared with the left arm of the stem; no such difference was observed for inner positions or for the stem as a whole (fig. 5).

**Fig. 6.—:**
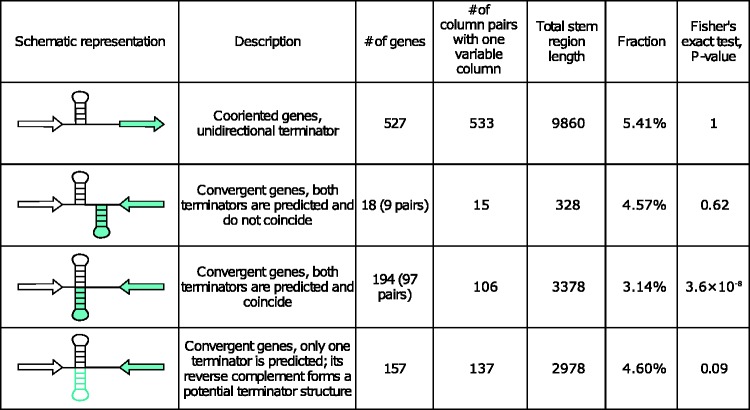
—Classes of bidirectional terminators. The last column represents the *P*-value of the Fisher's exact test, comparing the fraction of column pairs such that exactly one nucleotide was variable, in each category of terminators belonging to convergent genes to that in unidirectional terminators.

Some terminators can function in both directions, terminating transcription of convergent (end-to-end) genes transcribed in opposite directions from two different strands. Such terminators were more conserved than terminators of co-oriented (end-to-head) genes (fig. 6); however, no such difference was observed for nonoverlapping terminators of convergent genes (fig. 6), suggesting that the increased conservation stems from the same DNA segment functioning as a terminator for two genes, rather than from the convergent positioning of the genes *per se*.

### Rate and Pattern of WC Switches

Out of the 927 analyzed alignments, 208 carried at least one stem pair such that both nucleotides experienced at least one substitution, for a total of 302 column pairs. Because we were interested in a detailed analysis of such double switches, we subjected these alignments to an additional manual curation procedure. This left us with 202 alignments, including a total of 2,074 stem nucleotides and 286 column pairs with each nucleotide substituted at least once.

We then asked how the mutations at the two sites were distributed phylogenetically relative to one another, and, whenever possible, what the intermediate state had been. For this, in each of the 286 switching column pairs, we analyzed all possible pairs of blocks on the tree (fig. 1) carrying different WC pairs and separated by exactly two substitutions. Each pair of blocks resulting from this procedure corresponded to a single switch event (e.g., blocks 1–4, 2–4, and 3-4 in fig. 1). Forty-six column pairs carried no WC switches; that is, they contained no pairs of WC-blocks separated by two substitutions. Among the remaining 240 column pairs, 89 carried more than a single base pair switch, yielding a total of 482 switches (supplementary fig. S3, Supplementary Material online).

**Fig. 7.—:**
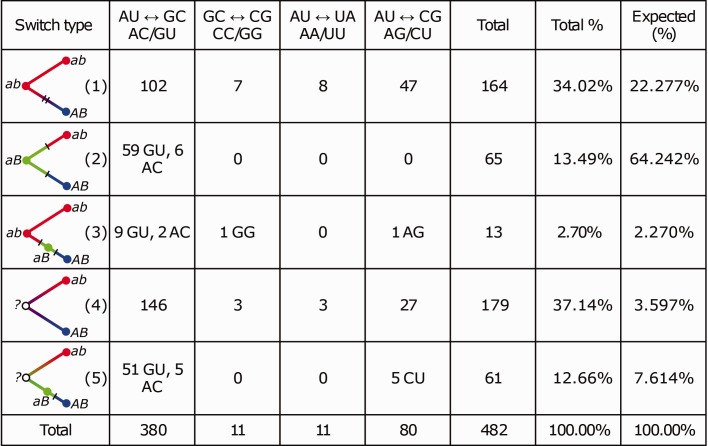
The distribution of observed base pair switches by five types defined in Fig. 2 (rows) and four possible pairs of intermediates (columns). The last column shows the expected distribution in permutations.

We categorized the observed switches based on the nucleotides identity, and also on whether the ancestral and the intermediate state could be inferred and on their phylogenetic position (fig. 2). In the resulting data (fig. 7), several features could be observed.

First, the WC pairs differed with regard to their propensity for switching. There are four possible kinds of switches between two WC pairs with unique intermediate groups: AU ⇊ GC (with intermediate states AC or GU), AU ⇊ CG (AG or UC), AU ⇊ UA (AA or UU), and GC ⇊ CG (CC or GG). The AU ⇊ GC switches constitute 79% of all switches, probably because one of the two intermediate variants, the GU wobble pair, is less deleterious. The reduced fitness loss associated with the GU pair is also confirmed by the data on switch types 2 and 3, where the intermediate state could be inferred with certainty. Here, 68 (89.5%) of all AU ⇊ GC switches involved GU as the intermediate, while only 8 (10.5%) involved AC as the intermediate (two-tailed binomial test, *P* = 5.6 × 10^−^^13^).

Furthermore, the propensity for switching differed between stem positions (supplementary fig. S4, Supplementary Material online). The frequencies of switches, and relative frequencies of switch types, differed between the external and internal positions of the stem. Switches were slightly more frequent at external positions: while such positions comprised 19% (391/2,074) of all stem positions, they carried 26% (126/482) of all switches (two-tailed binomial test, *P* = 9.0 × 10^−^^5^), and 39% (40/102) of switches that could not involve the GU intermediate (i.e., AU ⇊ CG, AU ⇊ UA or GC ⇊ CG; *P* = 1.6 × 10^−^^6^). Conversely, the rate of AU ⇊ GC switches possibly involving a GU intermediate was almost similar between the external and internal stem positions (23% of these switches occurred at external positions; *P* = 0.07).

Second, the phylogenetic distribution of mutations involved in switches was nonindependent. To show this, we compared the relative frequencies of the five switch types to those expected if the substitutions were independent of each other. First, we computed the terminal-to-intermediate ratio (TIR; Meer et al. 2010). This statistic is calculated as the ratio of the number of switches where the last common ancestor coincides with one of the two terminal states, to the number of those where it does not, and corresponds, in our analysis, to the number of switches of types 1 and 3, divided by the number of switches of type 2. When the LCA carries a terminal state (i.e., WC-state), both substitutions separating two blocks must have occurred in the same lineage; otherwise, each substitution has occurred in its own lineage. TIR equals 2.72 (177/65) in our dataset, while 0.38 (24.547/64.242) is expected based on permutation results (two-tailed binomial test, *P* = 1.0 × 10^−^^48^). Second, we computed the ratio of switches where the intermediate state was not observed, to those where it was, i.e., the number of switches of types 1 and 4, divided by the number of switches of types 2, 3, and 5. This ratio equals 2.47, while 0.35 is expected based on permutation results (two-tailed binomial test, *P* = 8.9 × 10^−^^96^). Therefore, both tests show that the intermediate states are observed less frequently than expected, implying that the two substitutions involved in a switch occur in rapid succession. The switches involving the GU intermediate are less rapid than other switches; indeed, among type 3 events where the phylogenetic distance between the two events could be estimated, the mutation from the GU intermediate occurs, on average, 0.019 substitutions per nucleotide after the mutation which established it, while this time is only 0.009 substitutions per nucleotide for other substitution pairs (Mann–Whitney *U* test, *P* = 0.044). Furthermore, TIR was higher (4.74) in the column pairs that each carried no more than three switches, and lower (1.18) in those column pairs that carried more than three switches, consistent with stronger selection against the intermediate variant in the more conservative site pairs, in line with a higher TIR in more conservative sites described previously in *Drosophila* proteins (Bazykin and Kondrashov 2012).

Dissimilarities between the strain tree and gene individual trees could result in erroneous inference of the phylogenetic distribution of events on the tree. To ask whether this substantially affects our conclusions, we tested whether our results hold under an alternative gene-based approach for tree reconstruction. For this, among the 202 genes where base pair substitutions were found, we selected a subset of 155 genes that had orthologs in the outgroup species, and reconstructed, for each such gene, a phylogenetic tree using orthologous gene sequences flanked by 1,000 or 2,000 nucleotides. Out of the 3,410 bipartitions observed in trees based on genes together with 2,000 flanking nucleotides, 2,247 (66%) were also observed in the strain tree; this fraction was higher for bipartitions with high bootstrap support in the gene tree (supplementary fig. S5, Supplementary Material online). The number of switches observed at a site in these trees remained the same in 131 out of 185 (71%) of site pairs; in 36 (19%) of sites pairs, the usage of gene trees decreased, and in 18 (10%) of site pairs, increased the number of switches. The discordance between the species tree and gene trees, and the resulting change in the number of observed switches, probably results from gene tree inference errors (Mendes et al. 2016). Indeed, the bootstrap support values for some of the branches for gene trees, including the branches between switching blocks, were low, making them unreliable for our switch analysis. Still, the TIR value remained similar (3.23 for 1,000 nt-flanks and 2.83 for 2,000 nt-flanks), implying that the conclusion that the two substitutions involved in a switch occur in rapid succession is not an artefact of tree reconstruction.

## Discussion

Here, we reconstruct the evolutionary history of rho-independent terminators of *B. cereus* genes, and study the forces that shape their evolution. We show that the stem structure of these terminators is highly conservative and maintained by strong natural selection. Nevertheless, the nucleotide sequence yielding this structure is dynamic, and we observe many paired mutational events rapidly substituting one WC pair for another.

Our analysis is subject to several limitations. First, our pairwise identity filtering ensures the high quality of analyzed alignments, but potentially biases the conservation upward, missing highly divergent terminators. However, only ∼17% of the terminators failed to pass the identity filter, and this filtering equally affects all compared categories of sites. Second, our analysis of substitutions in terminator stems is restricted to the reference terminator. Correct prediction of terminator stem boundaries is a challenging task because it requires understanding of the delicate balance between terminator stem energy and the energy of the DNA-RNA heteroduplex involving the poly-U tail. This problem might contribute to our observation of increased variability of external and, in particular, inner external stem positions. Therefore, we restricted our analysis to the predicted reference genome structure and did not analyze terminator elongation/shortening events. Third, our phylogenetic analysis assumes reliable inference of the tree shape and the phylogenetic distribution of events. However, factors such as horizontal gene transfer and insufficient phylogenetic power can lead to erroneous inferences. Our analysis of phylogenetic associations between substitutions using gene-level rather than genome-level trees suggests that this problem is unlikely to qualitatively affect our conclusions.

Given these caveats, we show that the substitutions in the terminator stems are rarer than those in terminator loops, consistent with the constraints imposed on each nucleotide in a pair by its complement. The vast majority of the single-nucleotide substitutions resulted in the GU wobble pair, consistent with the fact that its thermodynamic stability is comparable with that of WC pairs, and emphasizing its role as a stepping stone in the walks along the RNA fitness landscapes (Meer et al. 2010).

Nucleotide conservation depends on the position within the stem and on characteristics of the terminator. Switches at the external positions simply shorten the stem for one base pair and slightly lower its free energy while the internal switches might cause strong structure destabilization and dramatic energy drop. Consistently, positions deep within the stem are more conserved than outer and, in particular, inner positions of the stem. Terminators that terminate transcription of convergent genes are more constrained than unidirectional terminators, indicative of an increased selection.

Increased conservation implies that mutations disrupting interactions in terminator RNA stems are deleterious. Therefore, compensatory mutations should occur fast, and the intermediate state reducing fitness is observed rarely, as indicated by an elevated value of the TIR statistic. Substitutions involving GU intermediates occurred less rapidly than others, are more frequent in internal positions of RNA stems, and are more frequent than the alternative AC intermediate in the AU-GC switches, again consistent with weaker selection against the GU than against non-GU intermediates.

Nevertheless, substitutions through non-GU intermediates were also not instantaneous, as we observed such intermediates during base pair switches. This suggests that the fitness valleys involved in base pair switching are rather shallow. 

## Supplementary Material

Supplementary data are available at *Genome Biology and Evolution* online.

## Supplementary Material

Supplementary Data

## References

[evw295-B1] Assis R. 2014 Strong Epistatic Selection on the RNA Secondary Structure of HIV. PLOS Pathog. 10:e1004363. 25210786 10.1371/journal.ppat.1004363 PMC4161434

[evw295-B2] Bazykin GA. 2015 Changing preferences: deformation of single position amino acid fitness landscapes and evolution of proteins. Biol Lett. 11:20150315. 26445980 10.1098/rsbl.2015.0315 PMC4650171

[evw295-B3] Bazykin GA Kondrashov AS. 2012 Major role of positive selection in the evolution of conservative segments of Drosophila proteins. Proc R Soc B Biol Sci. 279:3409–3417. 10.1098/rspb.2012.0776 PMC3396909 22673359

[evw295-B4] Bazykin GA Kondrashov FA Ogurtsov AY Sunyaev S Kondrashov AS. 2004 Positive selection at sites of multiple amino acid replacements since rat–mouse divergence. Nature 429:558–562. 15175752 10.1038/nature02601

[evw295-B5] Ciampi MS. 2006 Rho-dependent terminators and transcription termination. Microbiology 152:2515–2528. 16946247 10.1099/mic.0.28982-0

[evw295-B6] Datta K von Hippel PH. 2008 Direct spectroscopic study of reconstituted transcription complexes reveals that intrinsic termination is driven primarily by thermodynamic destabilization of the nucleic acid framework. J Biol Chem. 283:3537–3549. 18070878 10.1074/jbc.M707998200 PMC2645038

[evw295-B7] De Hoon MJL Makita Y Nakai K Miyano S. 2005 Prediction of transcriptional terminators in Bacillus subtilis and related species. PLoS Comput Biol. 1:e25. 16110342 10.1371/journal.pcbi.0010025 PMC1187862

[evw295-B8] De Visser JAGM Krug J. 2014 Empirical fitness landscapes and the predictability of evolution. Nat Rev Genet. 15:480–490. 24913663 10.1038/nrg3744

[evw295-B9] Edgar RC. 2004 MUSCLE: multiple sequence alignment with high accuracy and high throughput. Nucleic Acids Res. 32:1792–1797. 15034147 10.1093/nar/gkh340 PMC390337

[evw295-B10] Elson JL, 2009 Pathogenic mitochondrial tRNA mutations - Which mutations are inherited and why? Hum Mutat. 30:E984–E992. 19718780 10.1002/humu.21113

[evw295-B11] Jukes TH Cantor CR. 1969 CHAPTER 24 - Evolution of protein molecules A2. In: Munro HN, Mammalian Protein Metabolism. New York: Academic Press p. 21–132.

[evw295-B12] Katoh K Toh H. 2008 Improved accuracy of multiple ncRNA alignment by incorporating structural information into a MAFFT-based framework. BMC Bioinformatics 9:212. 18439255 10.1186/1471-2105-9-212 PMC2387179

[evw295-B13] Kern AD Kondrashov FA. 2004 Mechanisms and convergence of compensatory evolution in mammalian mitochondrial tRNAs. Nat Genet. 36:1207–1212. 15502829 10.1038/ng1451

[evw295-B14] Kimura M. 1985 The role of compensatory neutral mutations in molecular evolution. J Genet. 64:7–19.

[evw295-B15] Kingsford CL Ayanbule K Salzberg SL. 2007 Rapid, accurate, computational discovery of Rho-independent transcription terminators illuminates their relationship to DNA uptake. Genome Biol. 8:R22. 17313685 10.1186/gb-2007-8-2-r22 PMC1852404

[evw295-B16] Li L. 2003 OrthoMCL: Identification of Ortholog Groups for Eukaryotic Genomes. Genome Res. 13:2178–2189. 12952885 10.1101/gr.1224503 PMC403725

[evw295-B17] López de Quinto S Martínez-Salas E. 2000 Interaction of the eIF4G initiation factor with the aphthovirus IRES is essential for internal translation initiation in vivo. RNA 6:1380–1392. 11073214 10.1017/s1355838200000753 PMC1370009

[evw295-B18] Lorenz R, 2011 ViennaRNA Package 2.0. Algorithms Mol Biol. 6:26. 22115189 10.1186/1748-7188-6-26 PMC3319429

[evw295-B19] Meer MV Kondrashov AS Artzy-Randrup Y Kondrashov FA. 2010 Compensatory evolution in mitochondrial tRNAs navigates valleys of low fitness. Nature 464:279–282. 20182427 10.1038/nature08691

[evw295-B20] Mendes FK Hahn Y Hahn MW. 2016 Gene tree discordance can generate patterns of diminishing convergence over time. Mol Biol Evol. 33:3299–3307. 27634870 10.1093/molbev/msw197

[evw295-B21] Mitra A Kesarwani AK Pal D Nagaraja V. 2011 WebGeSTer DB—a transcription terminator database. Nucleic Acids Res. 39:D129–D135. 20972211 10.1093/nar/gkq971 PMC3013805

[evw295-B22] Podgornaia AI Laub MT. 2015 Pervasive degeneracy and epistasis in a protein-protein interface. Science 347:673–677. 25657251 10.1126/science.1257360

[evw295-B23] Rousset F Pélandakis M Solignac M. 1991 Evolution of compensatory substitutions through G.U intermediate state in Drosophila rRNA. Proc Natl Acad Sci U S A. 88:10032–10036. 1946420 10.1073/pnas.88.22.10032 PMC52861

[evw295-B24] Stamatakis A. 2006 RAxML-VI-HPC: maximum likelihood-based phylogenetic analyses with thousands of taxa and mixed models. Bioinforma Oxf Engl. 22:2688–2690. 10.1093/bioinformatics/btl446 16928733

[evw295-B25] Stephan W. 1996 The rate of compensatory evolution. Genetics 144:419–426. 8878705 10.1093/genetics/144.1.419 PMC1207514

[evw295-B26] Talavera G Castresana J. 2007 Improvement of phylogenies after removing divergent and ambiguously aligned blocks from protein sequence alignments. Syst Biol. 56:564–577. 17654362 10.1080/10635150701472164

[evw295-B27] Tavaré S. 1986 Some probabilistic and statistical problems in the analysis of DNA sequences In: American mathematical society: lectures on mathematics in the life sciences. Vol. 17 Amer Mathematical Society, Providence, RI p. 57–86.

[evw295-B28] Thompson JD Higgins DG Gibson TJ. 1994 CLUSTAL W: improving the sensitivity of progressive multiple sequence alignment through sequence weighting, position-specific gap penalties and weight matrix choice. Nucleic Acids Res. 22:4673–4680. 7984417 10.1093/nar/22.22.4673 PMC308517

[evw295-B29] Yang Z. 1997 PAML: a program package for phylogenetic analysis by maximum likelihood. Comput Appl Biosci CABIOS. 13:555–556. 9367129 10.1093/bioinformatics/13.5.555

