# Supplementary material for: Compensatory Evolution of Intrinsic Transcription Terminators in Bacillus Cereus
Source: Genome Biol Evol. 2017 Feb 14;9(2):340–9. doi: 10.1093/gbe/evw295 (PMC5381666; doi:10.1093/gbe/evw295)
Supplement: Supplementary Data [file evw295_Supp.zip › S2_article_Safina_Mironov_Bazykin_supplementary_statistics.docx]

**Supplementary Information: Statistical calculations**

All calculations were performed in R.

**Figure 4:**

**AU pair**

Total number of column pairs is 3607 = 24 + 8 + 13 + 43 + 3217 + 3 + 171 + 6 + 70 + 52

Probability of A to change is P_A = (3 + 171 + 6 + 70 + 52)/3607 = 0.08372609

Probability of U to change is P_U = (24 + 8 + 13 + 43 + 52)/3607 = 0.03881342

Probability of two independent substitutions is P_AU = P_A * P_U = 0.003249696

Two-tailed binomial test p-value: 3.994591e-18.

> binom.test(52,3607,P_AU,a="t")$p.value

**GC pair**

Total number of column pairs is 4334 = 44 + 5 + 122 + 2 + 4012 + 53 + 6 + 13 + 77

Probability of G to change is P_G = (53 + 6 + 13 + 77)/4334 = 0.03437933

Probability of C to change is P_C = (44 + 5 + 122 + 2 + 77)/4334 = 0.05768343

Probability of two independent substitutions is P_GC = P_G * P_C = 0.001983118

Two-tailed binomial test p-value: 7.230742e-46.

> binom.test(77,4334,P_GC,a="t")$p.value

**GU pair**

Total number of column pairs is 333 = 40 + 1 + 2 + 44 + 205 + 9 + 3 + 5 + 24

Probability of G to change is P_G = (40 + 1 + 2 + 44 + 24)/333 = 0.3333333

Probability of U to change is P_U = (9 + 3 + 5 + 24)/333 = 0.1231231

Probability of two independent substitutions is P_GU = P_G * P_U = 0.04104103

Two-tailed binomial test p-value: 0.008004296.

> binom.test(24,333,P_GU,a="t")$p.value

**Figure 5:**

Fisher's exact test p-value for the tables provided in Figure 5 was calculated as follows:

external/internal, AU: 0.008126813.

> fisher.test(matrix(c(64,274,433,2784),2,2), a="t")$p.value

external/internal, GC: 2.518429e-05.

> fisher.test(matrix(c(93,152,1018,2994),2,2), a="t")$p.value

external/internal, GU: 0.6584997.

> fisher.test(matrix(c(20,84,45,160),2,2), a="t")$p.value

inner/outer: 2.337508e-06.

> fisher.test(matrix(c(63,114,813,683),2,2), a="t")$p.value

right arm/left arm: 0.001972936.

> fisher.test(matrix(c(19,44,813,813),2,2), a="t")$p.value

We calculate the Fisher's exact test p-value as the probability to obtain the given or more extreme number of variable columns, assuming that the two categories compared (external vs internal, inner vs outer or right arm vs left arm) are equally likely to contain variable columns.

**Figure 6:**

> fisher.test(matrix(c(15,328-15,533,9860-533),2,2), a="t")$p.value

> fisher.test(matrix(c(106,3378-106, 533,9860-533),2,2), a="t")$p.value

> fisher.test(matrix(c(137,2978-137, 533,9860-533),2,2), a="t")$p.value

**The GU intermediate is preferred over AC in types 2 and 3 switches:**

The probability to obtain 68 switches of types 2 and 3 involving the GU intermediate or more extreme observation out of the 76 switches of types 2 and 3 involving either GU or AC intermediates assuming that GU and AC are equally likely to be involved in a switch is the p-value of two-tailed binomial test: 5.632908e-13.

> binom.test(x=68, n=68+8, p=0.5, a="t")$p.value

**Switches are more frequent at external positions:**

For all switches: 8.981777e-05.

> binom.test(x=126,n=482,p=391/2074,alternative="t")$p.value

For switches not involving the GU intermediate: 1.569973e-06.

> binom.test(x=40,n=102,p=391/2074,alternative="t")$p.value

But not for AU ↔ GC switches: 0.06602678.

> binom.test(x=86,n=380,p=391/2074,alternative="t")$p.value

**TIR statistics:**

The probability to obtain 177 switches of types 1 and 3 or more extreme observation out of 242 switches of types 1, 2 and 3 under the null distribution obtained from permutations is the p-value of the two-tailed binomial test: 1.033987e-48.

> binom.test(x=164+13,n=164+13+65,p=(22.277+2.270)/( 22.277+2.270+64.242),a="t")$p.value

The probability to obtain 343 switches of types 1 and 4 or more extreme observation out of the total number of switches under the null distribution taken from permutations is the p-value of the two-tailed binomial test: 8.927824e-96.

> binom.test(x=164+179,n=164+13+65+179+61,p=(22.277+3.597)/(22.277+2.270+64.242+3.597+7.614),a="t")$p.value

**Mann-Whitney U-test showing that the switches involving the GU intermediate are slower than other switches:**

Mutations from the GU intermediate occur after, on average, 0.019 substitutions per nucleotide (average over: 0.0088346039, 0.0138604915, 0.0086418738, 0.0177169244, 0.0138604915, 0.03029123645, 0.02847255495, 0.0177169244, 0.03046808345).

Mutations from the non-GU intermediate occur after, on average, 0.009 substitutions per nucleotide (0.0105524255 for GG, 0.0086418738 for AC, 0.01248914235 for AG, 0.003997449 for AC).

Mann-Whitney U-test p-value: 0.04398211.

> wilcox.test(c(0.0088346039,0.0138604915,0.0086418738,0.0177169244,0.0138604915,0.03029123645,0.02847255495,0.0177169244,0.03046808345), c(0.0105524255,0.0086418738,0.01248914235,0.003997449), paired=FALSE)$p.value
