# Supplementary material for: Compensatory Evolution of Intrinsic Transcription Terminators in Bacillus Cereus
Source: Genome Biol Evol. 2017 Feb 14;9(2):340–9. doi: 10.1093/gbe/evw295 (PMC5381666; doi:10.1093/gbe/evw295)
Supplement: Supplementary Data [file evw295_Supp.zip › S1_article_Safina_Mironov_Bazykin_supplementary_figures.doc]

**Supplementary figures**

SF1. Reconstructed phylogeny with outgroup species (NC009674).

SF2. Distributions of the stem lengths (A), the number of uraciles in poly-U tract (B) and the loop lengths (C) in 927 selected terminators.

SF3. The distribution of the numbers of switches in 240 column pairs.

SF4. The distribution of observed base pair switches by five types defined in Fig. 2 (rows) and four possible pairs of intermediates (columns) at the external positions of the stems.

SF5. The distribution of bootstrap values for bipartitions observed in trees based on genes together with 2000 flanking nucleotides, with gray showing the distribution for bipartitions consistent with the strain tree.
